# Magnetic and structural properties of the solid solution CuAl_2(1−x)_Ga_2x_O_4_

**DOI:** 10.1038/s41598-021-89197-1

**Published:** 2021-05-31

**Authors:** T. J. Bullard, M. A. Susner, K. M. Taddei, J. A. Brant, T. J. Haugan

**Affiliations:** 1 UES, Inc., 4401 Dayton-Xenia Rd., Dayton, OH 45432 USA; 2 Aerospace Systems Directorate, Air Force Research Laboratory, Wright-Patterson AFB, Dayton, OH 45433 USA; 3 Materials and Manufacturing Directorate, Air Force Research Directorate, Wright-Patterson AFB, Dayton, OH 45433 USA; 4 Oak Ridge National Laboratory, 1 Bethel Valley Rd., Oak Ridge, TN 37830 USA

**Keywords:** Physics, Condensed-matter physics, Magnetic properties and materials

## Abstract

CuAl_2_O_4_ is a ternary oxide spinel with Cu^2+^ ions ($s=1/2$) primarily populating the A-site diamond sublattice. The compound is reported to display evidence of spin glass behavior but possess a non-frozen magnetic ground state below the transition temperature. On the other hand, the spinel CuGa_2_O_4_ displays spin glass behavior at ~ 2.5 K with Cu^2+^ ions more readily tending to the B-site pyrochlore sublattice. Therefore, we investigate the magnetic and structural properties of the solid solution CuAl_2(1-x)_Ga_2x_O_4_ examining the evolution of the magnetic behavior as Al^3+^ is replaced with a much larger Ga^3+^ ion. Our results show that the Cu^2+^ ions tend to migrate from tetrahedral to octahedral sites as the Ga^3+^ ion concentration increases, resulting in a concomitant change in the glassy magnetic properties of the solution. Results indicate glassy behavior for much of the solution with a general trend towards decreasing magnetic frustration as the Cu^2+^ ion shifts to the B-site. However, the $x=0.1$ and 0.2 members of the system do not show glassy behavior down to our measurement limit (1.9 K) suggesting a delayed spin glass transition. We suggest that these two members are additional candidates for investigation to access highly frustrated exotic quantum states.

## Introduction

Frustrated magnetic systems continue to be an area of intense interest due to the emergence of novel magnetic ground states ^1^. Among the many molecular geometries leading to magnetic frustration are the spinels (AB_2_X_4_, X = O, S, Sr). These materials are composed of a close packed *X* anion lattice containing alternating tetrahedral and octahedral sites that accommodate A^2+^ and B^3+^ cations, respectively. Population of either site with magnetic cations can lead to frustration. For instance, the octahedral sites create a corner-sharing tetrahedral pyrochlore network which can lead to geometric frustration if populated with magnetic moments with antiferromagnetic nearest neighbor (NN) exchange ^2–5^. The tetrahedral sites, which connect to form a diamond lattice, may also lead to frustration, as observed in FeSc_2_S_4_, MnSc_2_S_4_^6^, FeAl_2_O_4_^7^ and CoAl_2_O_4_^8^. In this case, frustration arises due to competing interactions between NN and next-nearest neighbors (NNN) on the lattice resulting in an interesting phase diagram^7^. In particular, if the exchange ratio ${J}_{\mathrm{NNN}}/{J}_{\mathrm{NN}}$ is above 1*/*8 and $s\le 1$ there is no thermal phase transition, but rather these compounds settle in a highly degenerate ground state associated with a spiral spin liquid^9,10^. For $s\ge 3/2$ a crossover to classical behavior occurs accompanied by a thermal phase transition to an ordered ground state. Therefore, spinels with spin at the quantum limit ($s=1/2$) populating the A-site are of particular interest in accessing highly degenerate quantum ground states.

While magnetic frustration may prevent a system from arriving at a well-ordered ground state, the addition of quenched disorder may lead to a glassy magnetic state such as a spin glass, where the energetics of the system are dominated by a rough free energy landscape, and hence slow relaxation dynamics. Despite intensive investigation, a consensus is still lacking as to the nature of the ground state and dynamics of the spin glass ^11^. One source of disorder in the spinel lattice leading to the spin glass state comes from cation mixing between sites. The fractional amount of B^3+^ atoms sitting on the A site is indicated by an inversion parameter $\eta$ such that the compound formula may be written as  $[{\mathrm{A}}_{(1-\upeta)} {\mathrm{B}_{\upeta}}]_{\mathrm{tet}}[{\mathrm{A}}_{\upeta} {\mathrm{B}}_{(2-\upeta)}]_{\mathrm{oct}}{\mathrm{X}}_{4}$. Spinels with $0<\eta <2/3$ are classified as “normal” spinels; otherwise, they are classified as “inverse” spinels.

It has recently been observed that the $x=0$ (CuAl_2_O_4_) member possesses indications of an exotic quantum ground state. Magnetic measurements reveal a high frustration factor $f=\frac{\left|{\Theta }_{cw}\right|}{{T}_{sg}}=67$ with a spin glass like cusp in the magnetic susceptibility at ${T}_{\mathrm{sg}}\sim 2$ K, a high Curie Weiss temperature ${\Theta }_{\mathrm{cw}}=-140$ K indicating a large antiferromagnetic exchange, and the onset of a metastability between the FC and ZFC spectra^12^. However, muon spin resonance (µSR) reveals a non-frozen state with significant dynamic spin fluctuation below the spin glass-like freezing transition. Site dislocation plays a role in introducing glassy behavior, but nevertheless spin fluctuations associated with the liquid state persists^13^. Prior investigation of this end member show that there is some inter-site mixing, with $\eta = 0.35 - 0.4$, indicating that the Cu^2+^ ions primarily populate the diamond sublattice^14^.

At the other end of the solid solution, CuGa_2_O_4_ is reported as an inverse spinel with the copper atoms primarily populating the pyrochlore sublattice $(\eta$ = 0*.*85) ^15^. Spin glass behavior is reported, with an onset at $3.8$ K indicated by µSR and a cusp in the susceptibility at $2.5$ K. It is argued that, in addition to the pyrochlore geometry, Jahn–Teller distortion plays a role in the frustration by introducing a random anisotropic exchange between Cu^2+^ spins^16^. Both end members of this system are also reported to have a non-zero zero-point entropy, characteristic of frustrated systems, and on the order of the predictions from the Sherrington-Kirkpatrick XY spin glass model^17^.

In this work we examine the magnetic and structural properties of the entire solid solution of this chemical system. We find magnetic behavior consistent with either an insulating spin glass, or a cluster glass where domains of net spin act as the magnetic entity of interest^18^. For $x=0$, we find a high frustration value similar to what is reported above^12^. We also show that the behavior of the ac susceptibility for the $x=0$ member fits a typical dynamical phase transition model for spin glasses. For $x>0.2$, as Al is replaced with Ga, we observe a decrease in the frustration in the system which correlates with Cu^2+^ ions shifting to the octahedral sites. However, for the $x = 0.1$ and $0.2$ compositions, we detect no peak in the ac susceptibility. We speculate that glassy behavior for these members occurs below the minimum accessible temperature of our apparatus; this would suggest a high degree of magnetic frustration, and therefore of interest for further investigation.

## Results

### Structural characterization

**Figure 1:**
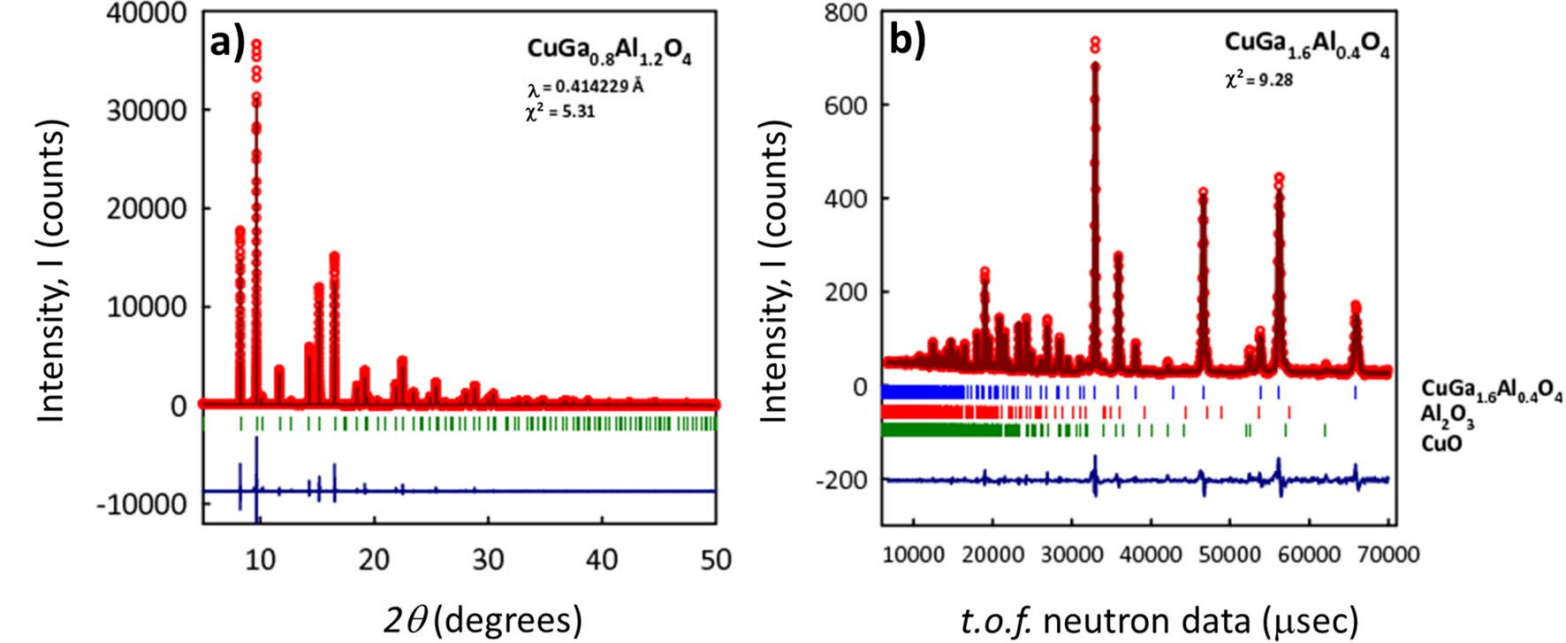
Examples of **(a)** synchrotron and **(b)** neutron diffraction data along with Rietveld refinements.

**Figure 2:**
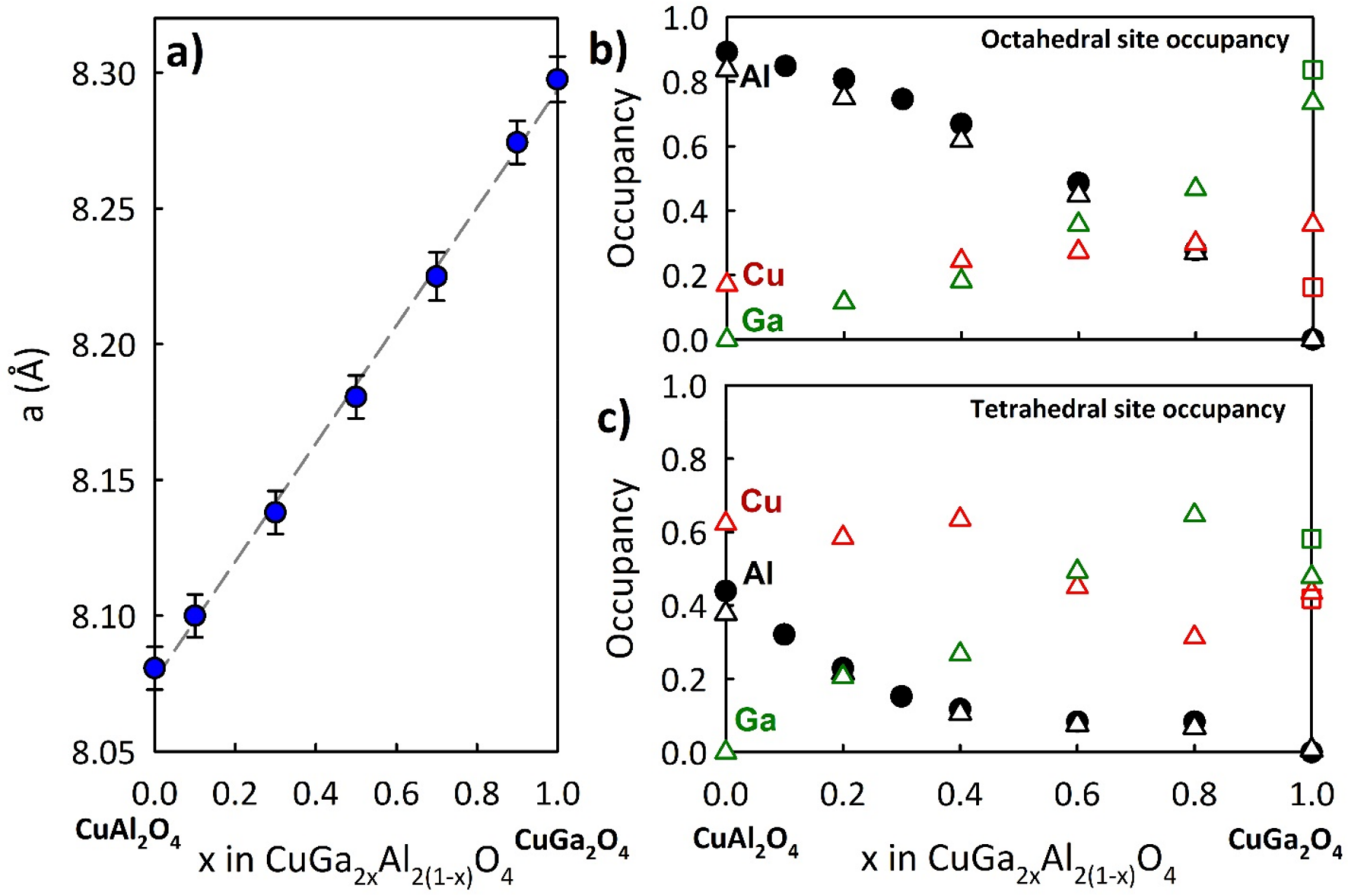
**(a)** Lattice parameter versus Ga fraction x. Results show linear behavior following Vegard’s law. Occupancy for the octahedral **(b)** and **(c)** tetrahedral sites. Circles indicate data derived from the synchrotron diffraction measurements; triangles indicate neutron diffraction derived data. Uncertainty for the results in **(b,c)** is $\sim 5\%$.

Room temperature synchrotron X-ray diffraction results show a well formed CuAl_2(1−x)_Ga_2x_O_4_ phase for the entire solid solution. As an example, the results for the $x=0.4$ member are shown in Fig. 1a. Small amounts of residual CuO are visible in the $x=0$ and $x=1$ members. All the members are found to be in the *Fd* $\overline{3 }m$ space group and obey Vegard’s law as shown in Fig. 2a indicating that the Ga^3+^ is taken up into the lattice replacing the Al^3+^. We also obtained neutron diffraction data for the even members of the solution. We present results for the $x=0.8$ member on the right in Fig. 1b along with its refinement fit. Refinement for CuAl_2_O_4_ agrees well with published results in terms of lattice parameter and chemical occupancy^14,15^ with $\eta =0.38$. On the other hand CuGa_2_O_4_ differs from prior reported results^15^ with $\eta =0.47$.

For the intermediate members of the solution, determination of the occupancy with X-ray diffraction required a two-step process due to challenges in discriminating between nearly isoelectronic ions such as Cu^2+^ and Ga^3+^. This difficulty was exacerbated by having to determine simultaneously the location of three cations. To deconvolute the metal occupancies, the location of the Al^3+^ cation was first determined for all compositions by performing a refinement on our high-resolution X-ray data with Ga^3+^ acting as a surrogate for Cu^2+^. Using these values as a starting point, we repeated the refinement process with the neutron diffraction data. The difference in coherent neutron scattering cross section was great enough to allow a determination of the occupancies of the Cu^2+^ (${\sigma }_{c}=7.485$) and Ga^3+^ (${\sigma }_{c}=6.675$) cations. In the final step, all cation occupancies were allowed to simultaneously refine. We obtained similar Al^3+^ occupancies for both the synchrotron and neutron data as shown in Fig. 2b,c. We further note that we also obtained similar Al^3+^ occupancies by using Cu^2+^ as the surrogate for Ga^3+^ (not shown).

The occupancies for the tetrahedral and octahedral sites are displayed in Fig. 2b,c. For the $x = 0$ member, Al^3+^ has a strong tendency to occupy the octahedral sites. The Cu^2+^ ions prefer, but do not completely populate the tetrahedral sites, as previously reported^14^. We note that structural results reported in the literature show no structural distortion or magnetic long range order down to 0.4 K for the $x=0$ member^12^. As $x$ increases (i.e., Al^3+^ is replaced by Ga^3+^) the Cu^2+^ ions are partially shift to the octahedral sites.

### High temperature susceptibility

**Figure 3:**
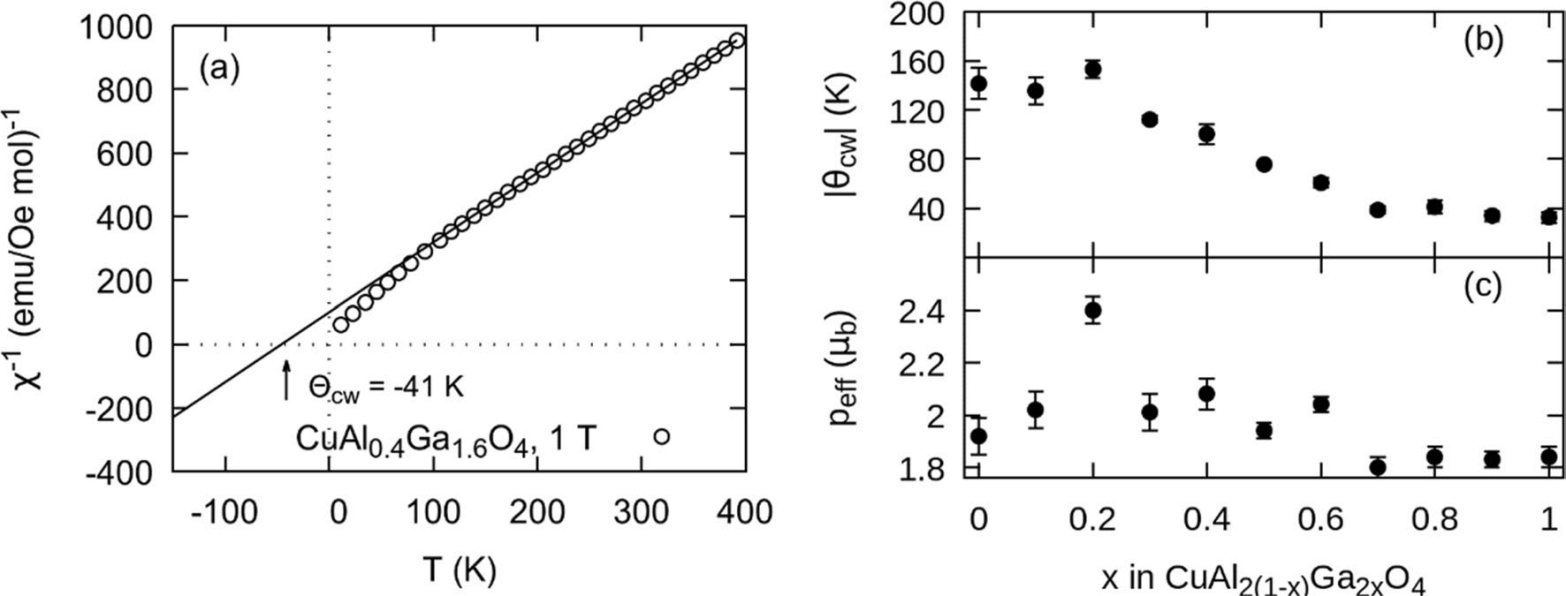
**(a)** Curie–Weiss behavior for the $x=0.8$ member of the solid solution; **(b)** extracted Curie Weiss-temperature, ${\Theta }_{cw}$; and **(c)** effective magnetic moment, ${p}_{eff}$ are extracted from the Curie–Weiss law for each member.

We describe all high temperature paramagnetic behavior for all members of the solid solution with the Curie–Weiss Law, ${\left(\chi -{\chi }_{0}\right)}^{-1}=\frac{3{k}_{B}(T-{\Theta }_{\mathrm{cw}})}{{A}_{v}{{p}_{\mathrm{eff}}}^{2}{{\mu }_{b}}^{2}}$. In this equation ${\chi }_{0}$ is the temperature independent portion of the magnetic susceptibility,${A}_{v}$ is Avogadro’s number, ${k}_{B}$ is the Boltzmann constant, ${\Theta }_{\mathrm{cw}}$ is the Curie–Weiss temperature, and ${p}_{\mathrm{eff}}$ is the effective magnetic moment in units of Bohr magnetons,${\mu }_{b}$. A characteristic result for the $x = 1$ member is plotted in Fig. 3a. The inverse susceptibility begins to deviate from linear paramagnetic behavior around 130 K similar to prior reported results^16^. ${\Theta }_{\mathrm{cw}}$ for the entire solid solution is reported in Fig. 3b and ${p}_{\mathrm{eff}}$ in Fig. 3c. The solid solution is found to have high temperature effective magnetic moments ranging between 1*.*8 and 2*.*4 ${\mu }_{b}$ with a maximum at $x = 0.2$. Typical observed values for materials with Cu^2+^ range from $1.7$ to $2.2{ \mu }_{b}$; similar to the theoretical value ${p}_{\mathrm{eff}}=g\sqrt{s(s+1)}=1.7 {\mu }_{b}$ ^19^. All Curie Weiss temperatures are found to be negative, indicating a short-range antiferromagnetic exchange interaction between spins. Concomitant with the Cu^2+^ cations repositioning to the octahedral sites (cf. Fig. 2), we find that ${\Theta }_{\mathrm{cw}}$ decreases in magnitude for $x>0.2$.

A similar nonlinear decrease in ${\Theta }_{\mathrm{cw}}$ has been observed in the system CoAl_2(1−x)_Ga_2x_O_4_. This decrease is associated with an increasing bond length that is geometrically necessary with the presence of the larger Ga atoms, and in turn decreases the exchange strength between the Co ions. Further, a linear decrease in ${\Theta }_{\mathrm{cw}}$ with bond distance is observed when comparing the end members of this Co solid solution and the well-ordered A-stie compounds Co_3_O_4_ and CoRh_2_O_4_. This suggests that the non-linearity in ${\Theta }_{cw}$ versus $x$ for both CoAl_2(1−x)_Ga_2x_O_4_ and CuAl_2(1−x)_Ga_2x_O_4_ is due to intersite mixing^20^. To a lesser extent we also see a decrease in ${p}_{\mathrm{eff}}$ after $x = 0.2$. Increasing orbital quench on the octahedral sites may play a role.

### DC susceptibility

**Figure 4:**
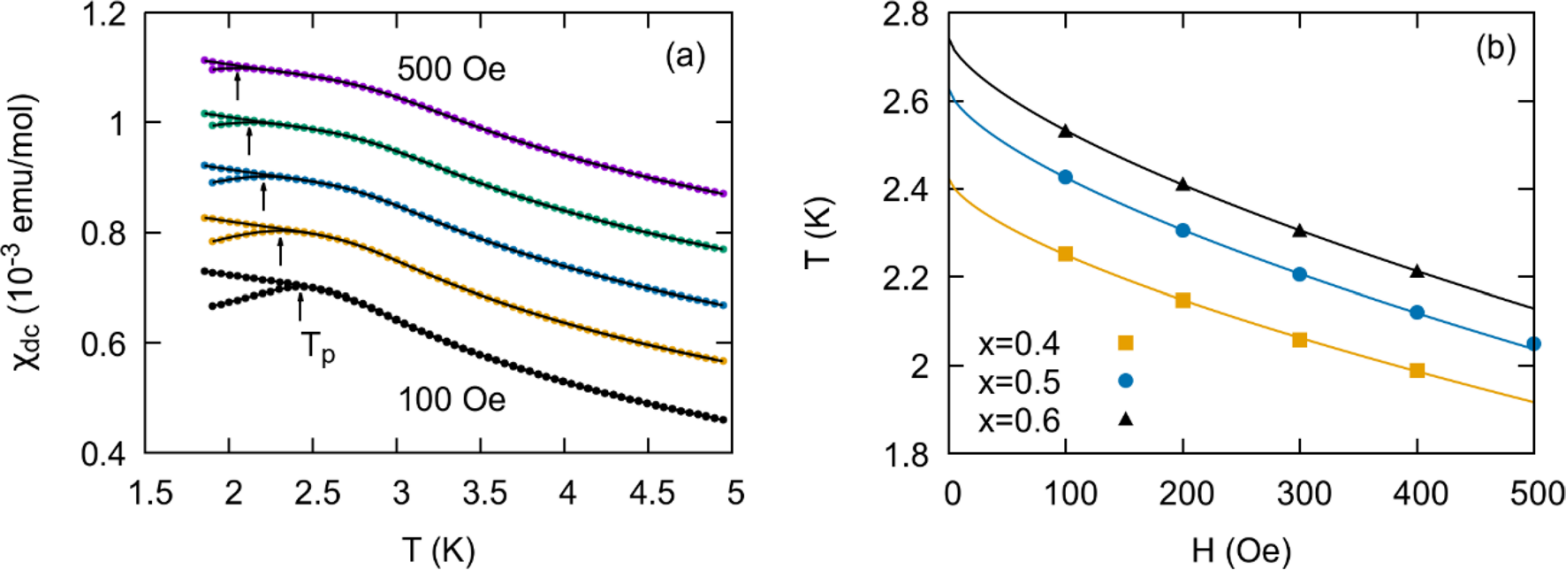
**(a)** FC-ZFC curves for the $x=0.5$ member of the solid solution with dc fields ranging from 100 to 500 Oe. Arrows indicate the peak in the ZFC curve. Curves have been shifted along the y-axis for comparison, **(b)** ${T}_{p}$ as a function of applied field $H$ for three of the solution members. Lines indicate fits to Eq. (1).

We have obtained FC-ZFC dc magnetic susceptibility curves in magnetic fields ranging from 100 to 500 Oe for all solid solution members. We observe bifurcation for members $x=0$ and $x\ge 0.3$. Typical results are plotted in Fig. 4a. Above 500 Oe the bifurcation is suppressed in the detectable range of measurement. We note that the FC curve does not flatten below the bifurcation temperature as observed in many spin glasses. This may suggest a portion of the spins are free from the glassy dynamics.

The de Almeida-Thouless (AT) phase boundary separating the spin glass state from the paramagnetic state is proposed in the Sherrington-Kirkpatrick (SK) mean field Ising spin glass model^21^, and is described as a power law of the form

1 $${T}_{irr}\left(H\right)={T}_{irr}\left(0\right)\left(1-C{H}^{\frac{2}{\Phi }}\right)$$

where ${T}_{\mathrm{irr}}$ is the irreversibility temperature, $H$ is the externally applied dc field,* C* is a constant, and $\Phi =3$. In Heisenberg systems a second boundary, the Gabay-Tolouse (GT) line, is associated with the freezing of the longitudinal components of the magnetic moment and has a predicted exponent of $\Phi =1$1 ^22^ . Non-mean field predictions allow $\Phi$ to vary, and experimental results capturing deviation from mean field predictions have been observed in systems such as GdAl^23^, CuMn_0.25_^24^ , and Cr_0.5_Fe_0.5_Ga^11^.

In our measurements, the FC-ZFC separation temperature $({T}_{\mathrm{irr}})$ is indistinguishable from the ZFC peak temperature $\left({T}_{p}\right)$. Therefore, we track ${T}_{p}$ as a function $H$ and find that best fit values for $\Phi$ reported in Table 1 range from 2.9 to 4.6. We do not observe any clear trend for $\Phi$ with material composition. Though $\Phi$ tends to be slightly larger than the mean field value, best fit curves with a fixed exponent of $3$ also yield statistically reasonable results. Typical results are displayed in Fig. 4b. Additional measurements below 100 Oe are required for accurate estimates for ${T}_{\mathrm{irr}}\left(0\right)$. Evidence for a GT line is not observed at these magnetic field values.

**Table 1:** Exponent describing the phase boundary in the T-H diagram separating paramagnetic and glassy behavior (Eq. 1). The exponent was obtained using both dc and ac susceptibility measurements (see subsection “Fixed $${H}_{ac}")$$.

X	${\Phi }_{dc}$	${\Phi }_{ac}$
0.3	–	5 ± 3
0.4	3.3 $\pm$ 0.6	3.9 ± 0.7
0.5	3.0 $\pm$ 0.4	4.4 ± 0.4
0.6	4.0 $\pm$ 0.5	3.5 ± 0.4
0.7	2.9 $\pm$ 0.1	3.3 ± 0.5
0.8	3.1 $\pm$ 0.2	3.8 ± 0.3
1.0	4.6 $\pm$ 0.4	3.2 ± 0.4

### Slow relaxation and memory

**Figure 5:**
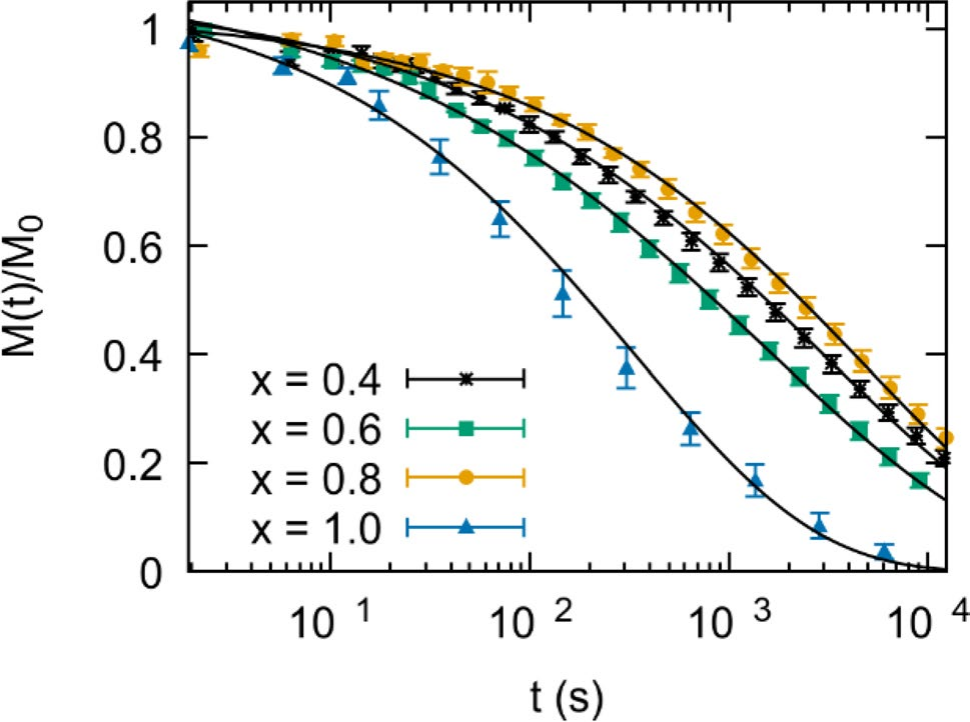
FC magnetization vs time at 1.9 K after a 200 Oe dc field has been removed. ${t}_{w}=7200$ s. Black lines indicate fits to Eq. (2)*.*

In Fig. 5 we show the time dependent magnetization for solution members $x=0.4, 0.6, 0.8$, and $1.0$. Each sample was field cooled below ${T}_{p}$ down to 1.9 K in the presence of a 200 Oe field. After a wait time ${t}_{w}=7200$ s the field was set to zero at a rate of 5 Oe/s. As shown in the graph we observe slow relaxation curves, and in all cases the data is reasonably fit to a stretched exponential of the form

2 $$M\left( t \right) = M_{0} exp( - (t/\tau^{\prime})^{1 - n} + M_{1}$$

where *M*_0_ is the FC magnetization and ${\tau }^{^{\prime}}$ is the characteristic relaxation time. An additional offset ${M}_{1}$ was included for an improved quality of fit. However, ${M}_{1}\ll$ ${M}_{0}$ and was neglected in the fits shown in Fig. 5a. Such a model has been used to describe slow relaxation results for a number of glassy systems including spin glasses ^25,26^. The exponent $1-n$ ranges from 0*.*39 to 0.68 with no clear trend in composition. The percolation model for spin glasses puts a bound on the exponent such that $\frac{1}{3}<1-n<1$ ^25^. The value ${\tau }^{^{\prime}}$ ranges from 200 s for the $x = 1$ member to 2200 s for $x = 0.8$. Additional investigation is required to show if varying ${t}_{w}$ leads to aging.

**Figure 6:**
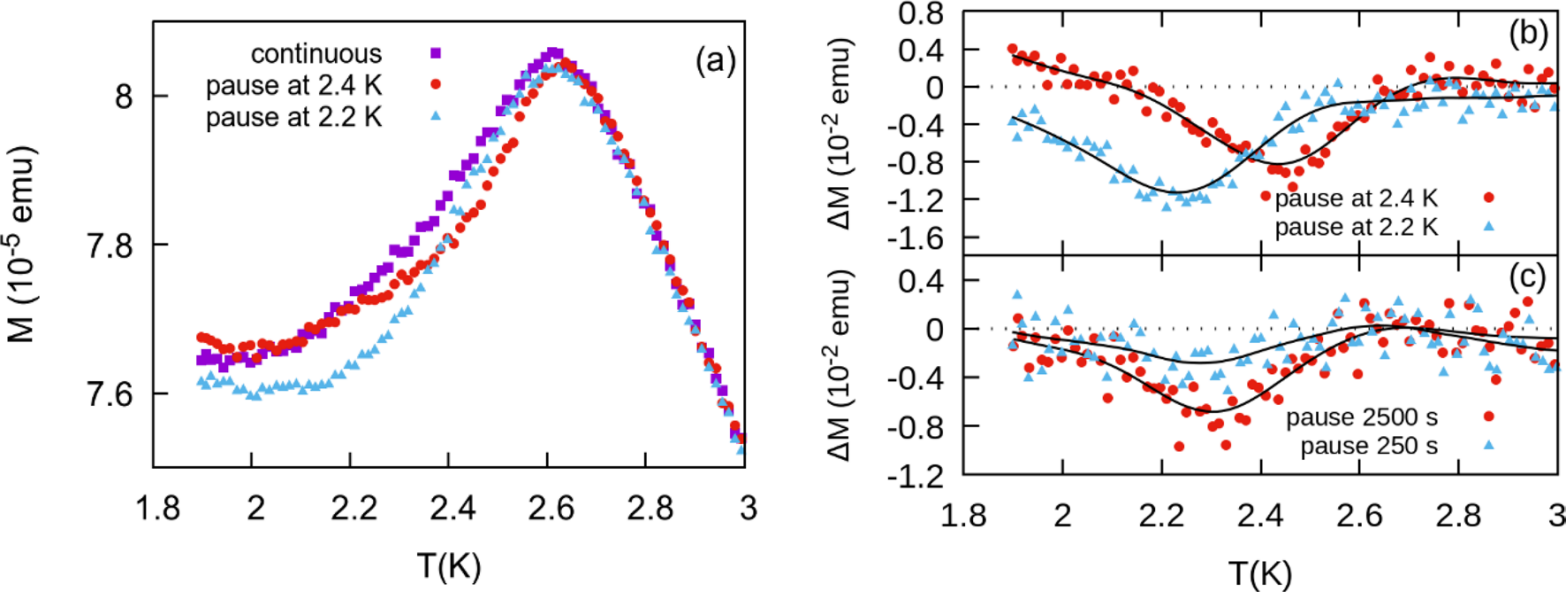
**(a)** ZFC curves for $x= 0.8$ at $H=50$ Oe. On separate measurements the system is paused at 2.2 and 2.4 K. **(b)** $\Delta M$ for the curves in **(a)**. **(c)** $\Delta M$ for ZFC curves paused at 250 and 2500 s at 2.3 K. Black lines are included as a guide for the eye.

Memory effects are also well known in magnetic glassy systems. We have performed two sets of memory experiments on the $x=0.8$ member of the solid solution. In the first set, we pause the cooling of the system at 2.2 K, and on a separate run at 2.4 K, as the temperature is cooled to 1.9 K in zero field. We then apply a dc field of 50 Oe and measure the magnetization as the temperature is increased. Results in Fig. 6a show a deviation from the continuous (unpaused) ZFC temperature sweep. To better demonstrate the memory effect, we also plot the difference between the unpaused ZFC curve and each paused curve such that

3 $$\Delta M=\frac{{M}_{ZFC(pause)}-{M}_{ZFC}}{{M}_{ZFC}}.$$

$\Delta M$ shows minima at the two temperatures at which the system was paused in Fig. 6b. For the second set of experiments, we paused the system at 2.3 K for 250 s and 2500 s. Again $\Delta M$ shows minima at 2.3 K in Fig. 6c with the longer pause time showing the larger effect. Results clearly indicate memory effects and hence the presence of a glassy magnetic state below ${T}_{p}$ for $x=0.8$.

### AC susceptibility

#### Fixed ${H}_{ac}$

**Figure 7:**
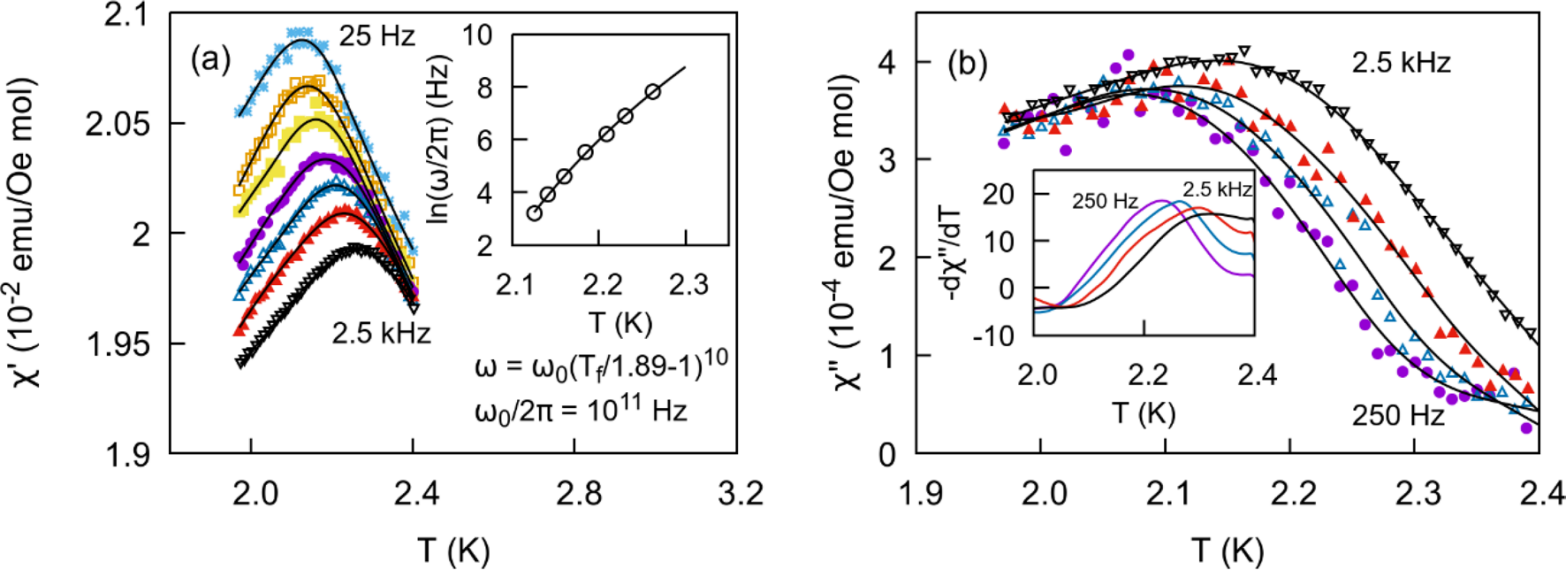
Temperature dependence of the real **(a)** and the imaginary **(b)** parts of the ac susceptibility for different frequencies for the $x=0$ member of the solid solution. Frequency vs. freezing temperature is fit to a dynamical power law in the inset of **(a)**. The inset in **(b**) shows the evolution of $\frac{d{\chi }^{{{\prime \prime}}}}{dT}$ as a function of frequency.

Further insight into the dynamic properties of glassy magnetic systems including spin glasses can be obtained through ac susceptibility measurements. We performed measurements on all members of the CuAl_2(1−x)_Ga_2x_O_4_ solid solution at frequencies ranging from 25 Hz to 2.5 kHz with an excitation field ${H}_{\mathrm{ac}}=3$ Oe. The real part of the susceptibility ${\chi }^{{\prime}}$ shows a peak for $x=0$ (Fig. 7a) and $x\ge 0.3$. The corresponding imaginary part of the susceptibility ${\chi }^{{{\prime \prime}}}$ (Fig. 7b) displays a maximum at temperatures lower than the peak in ${\chi }^{^{\prime}}$ followed by an inflection point at higher temperatures. Both show a frequency dependent trend as expected for glassy systems with the peak/inflection point shifting to higher temperatures as the frequency increases. The relative shift in temperature per frequency decade may be quantified as

4 $$K=\frac{\Delta {T}_{f}}{\left[{T}_{f}*\Delta {log}_{10}\left(\frac{\omega }{2\pi }\right)\right]}$$

where ${T}_{f}$ is the temperature of the peak in ${\chi }^{{\prime}}$, $\omega /2\pi$ is the excitation frequency, and ∆ indicates the change between frequencies. ${T}_{f}$ is estimated using a spline curve to locally smooth the data. As indicated in Table 2, $K$ ranges from 0*.*03 to 0*.*04. Such values are an order of magnitude larger than canonical metallic spin glasses but smaller than insulator spin glasses (0.06–0.08)^27,28^. For a superparamagnet,  $K > 0.2$^27^.

**Table 2:** x indicates the fraction of Ga in CuAl_2(1-x)_Ga_2x_O_4_. Columns 2–5 are parameters derived from Eqs. (4) and (5). ${H}_{ac}=3\mathrm{ Oe}$.

x	$K$	${\tau }_{0}$ $( \mathrm{s})$	${T}_{sg} (\mathrm{K})$		$z\upsilon$
0	0.037	${10}^{-11\pm 0.5}$	1.89 ± 0.05		10 ± 2
0.4	0.034	${10}^{-11\pm 2}$	2.3 ± 0.1		9 ± 4
0.6	0.032	${10}^{-13\pm 0.5}$	2.3 ± 0.1		17 ± 4
0.8	0.028	${10}^{-12\pm 0.5}$	2.57 ± 0.03		10 ± 1
1.0	0.030	${10}^{-14\pm 2}$	2.3 ± 0.2		16 ± 9

The frequency dependent shift in temperature can be described in terms of a classical phase transition, where the correlation time diverges as a power law such that $\tau \propto {\xi }^{z}$. $\xi$ is the correlation length and *z* is the dynamical scaling exponent. The correlation length is in turn related to the reduced temperature $\xi \propto {\left(\frac{{T}_{f}-{T}_{sg}}{{T}_{sg}}\right)}^{-\nu }$ where $\nu$ is the correlation length critical exponent. The correlation time near the critical point is then $\tau ={\tau }_{0}{\left(\frac{{T}_{f}-{T}_{sg}}{{T}_{sg}}\right)}^{-z\nu }$ where ${\tau }_{0}$ is the characteristic magnetic moment spin-flip time. Associating the excitation frequency with the inverse of the correlation time we rewrite the relationship as

5 $$\omega ={\omega }_{0}{\left(\frac{{T}_{f}-{T}_{sg}}{{T}_{sg}}\right)}^{z\nu }.$$

The susceptibility should then diverge as the temperature approaches the spin glass temperature ${T}_{s\mathrm{g}}$ at zero frequency. From these temperatures we obtain parameters from a 3-variable fit to the equation $\mathrm{ln}\omega =\mathrm{ln}{\omega }_{0}+z\nu \mathrm{ln}\left(\frac{{T}_{f}}{{T}_{sg}}-1\right)$. Results for frequencies above 2.5 kHz are not included in the fit due to evidence of Joule heating from eddy currents induced by the ac field. We also find that results below 25 Hz are too noisy to obtain a precise estimate of the peak location. Fit parameters for the even members of the solution are reported in Table 2. Values obtained for ${\tau }_{0}$ derived from these fittings are accurate to only the nearest order of magnitude. Nevertheless the values fall within the range for canonical spin glasses (10^–12^–10^–14^ s)^25,29,30^ while values as low as 10^–7^ have been reported for insulator spin glasses such as Eu_0.4_Sr_0.6_S^31^ and cluster glasses such as Nd_5_Ge_3_^32^. Values for cluster glasses range from 10^–6^ to 10^−11^ s^28^. We were also able to estimate the dynamical exponent $z\nu$ from the data. Typical values for $z\nu$ range from 4 to 12 for glassy magnetic systems ^27^. However, results as high as 13 have been reported in a number of cases ^33–36^ while 14 has been associated with the random field Ising magnet^37^. Results obtained in this study range from 9 to 17; however, we hesitate to draw any strong conclusions due to the large uncertainty associated with these results, necessitating a more careful approach in subsection “Variable H_ac_^”^.

${\chi }^{{{\prime \prime}}}$ displays a similar shift in temperature with increasing frequency. The maximum of the slope ${\left(\frac{d{\chi }^{{{\prime \prime}}}}{dT}\right)}_{\mathrm{max}}$ has been associated with ${T}_{f}$^27,38^, but in our data we clearly see the maximum occurring at a slightly higher temperature than the peak in ${\chi }^{^{\prime}}$. Due to a much smaller signal no frequency dependent trends could be extracted from this data. Plots of ${\chi }^{{{\prime \prime}}}$ and $\frac{d{\chi }^{{{\prime \prime}}}}{dT}$ versus T for the $x=0$ member of the solution are displayed in Fig. 7b.

**Figure 8:**
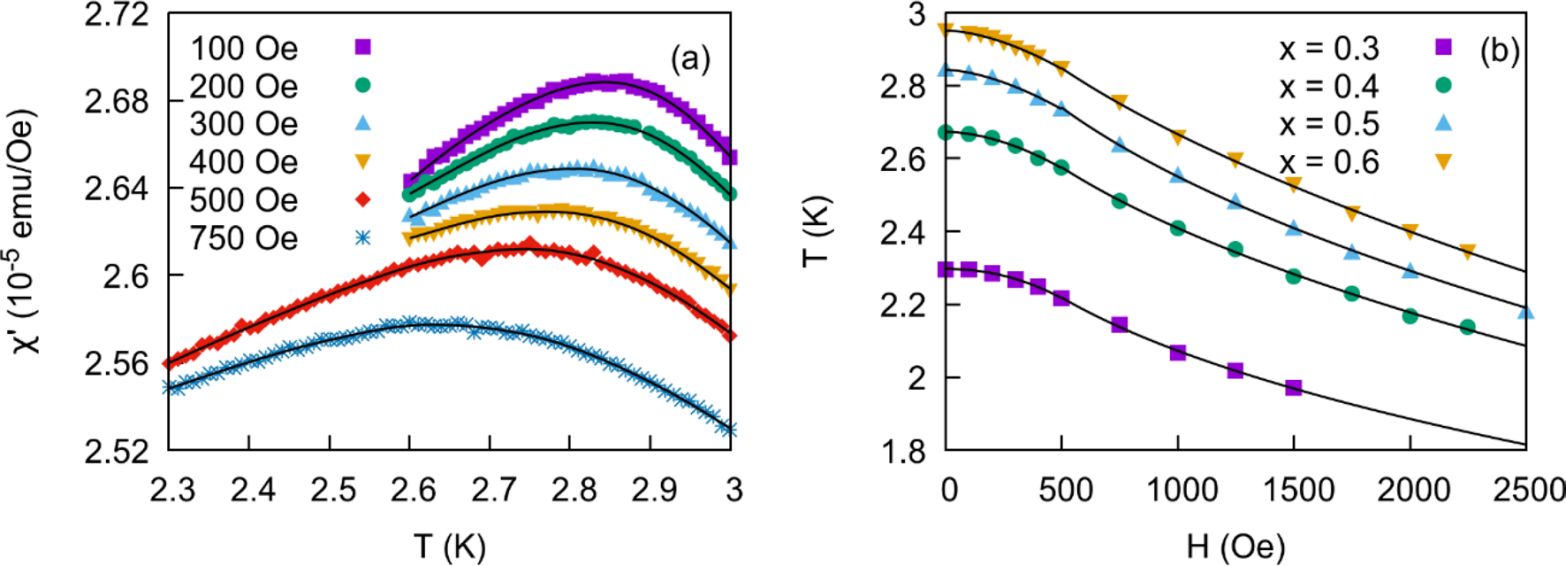
**(a)** ${\chi }^{^{\prime}}$ versus $T$ for $x=0.5$. Peak in ${\chi }^{^{\prime}}$ broadens and shifts to lower temperatures in higher external dc fields. **(b)** ${T}_{f}$ vs H shows AT like behavior above 500 Oe for all members of the solid solution.

We also examined the shift in ${T}_{f}$ in the presence of an external dc field. Results for $x=0.5$ are shown in Fig. 8a. As the field increases the peak both broadens and shifts to lower temperatures, as has been observed for many magnetic glassy systems. Results for the entire solid solution were obtained at $\frac{\omega }{2\pi }=$ 1 kHz and ${H}_{\mathrm{ac}}=3$ Oe. As shown in Fig. 8b, above 500 Oe we observe an AT like power law trend similar to Eq. (1). Best fits for $\Phi$ are reported in Table 1 and vary from 3.2 to 5; all values are higher than the mean field value of 3. Non-mean field analysis on a number of spin glasses show $\Phi$ varying from 3.2 (random anisotropic $\alpha -$ DyNi^39^) to 5 (CuMn^40^), suggesting that values obtained for this solid solution are reasonable in a spin glass framework. As a benchmark, a value of 3.8 is obtained for the cluster glass Cr_0.5_Fe_0.5_Ga^11^. For $H < 500$ Oe we observe a crossover from AT like behavior to $T\propto {H}^{2}$. However, we do not believe this is associated with the GT line. Similar behavior is observed in ac susceptibility measurements of insulating spin glass Eu_0.4_Sr_0.6_S^41^, and is due to shortened relaxation times (higher frequencies) in low fields. The behavior diminishes for lower frequencies for this solid solution (not shown) and in published results (e.g. ^42^).

#### Variable H_ac_

To decrease the uncertainty in the estimates obtained in the dynamical scaling analysis we repeated the measurements while varying the magnitude of the ac driving field. We chose the magnitude for each frequency such that it maximizes the ac signal while also avoiding Joule heating. This careful approach also extends the frequency range over which models may be fit to the data. Results are reported for comparison in Table 3.

**Table 3:** Same parameters as those in Table 2 with ${H}_{ac}$ varying as indicated in Fig. 9a.

x	$K$	${\tau }_{0}$ $(\mathrm{s})$	${T}_{sg} (\mathrm{K})$	$z\upsilon$
0	0.031	${10}^{-11\pm 0.3}$	1.82 $\pm$ 0.06	13 ± 2
0.4	0.031	${10}^{-12\pm 0.4}$	2.19 $\pm$ 0.09	13 ± 3
0.6	0.027	${10}^{-12\pm 0.3}$	2.46 $\pm$ 0.05	13 ± 1
0.8	0.026	${10}^{-12\pm 0.5}$	2.52 $\pm$ 0.05	12 ± 1
1.0	0.026	${10}^{-12\pm 0.8}$	2.47 $\pm$ 0.07	11 ± 2

**Figure 9:**
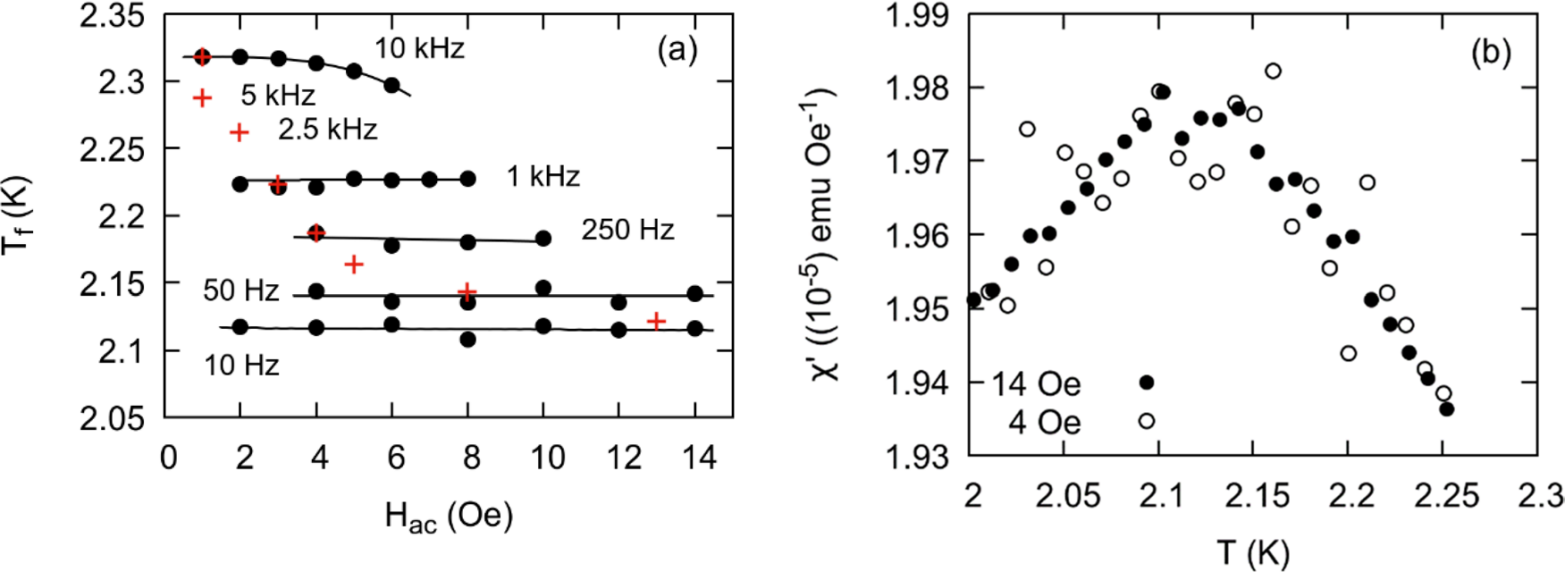
**(a)** Black dots indicate ${T}_{f}$ as a function of ${H}_{ac}$ at different frequencies. Results are shown for x = 0. Red crosses indicate ${H}_{ac}$ magnitudes chosen for different frequencies for reported results in Tables 2, 3, and 4. Values were chosen to maximize measurement signal and minimize a nonlinear response. Black lines are introduced as a guide for the eye. (b) Peak in ${\chi }^{^{\prime}}(T)$ at 10 Hz and driving fields of 4 and 14 Oe.

Such ac measurements are typically performed with ${H}_{\mathrm{ac}}$ held constant across all frequencies. The excitation magnitude is chosen to be small so as to avoid introducing nonlinearity to the measurement ^18,43^. However, we demonstrate that the excitation field can be varied to maximize signal to noise while leaving ${T}_{f}$ unaffected. We demonstrate this in Fig. 9a where we measured ${T}_{f}$ as a function of excitation amplitude from 10 Hz to 10 kHz for $x=0$. We also report the values of ${H}_{\mathrm{ac}}$ selected for the measurements presented in this section. Below 1 kHz we observe no significant shift in freezing temperature for the range of dc fields chosen, while at 10 kHz we observe a deviation to lower temperatures at higher fields. Further, we observe no significant change in the shape or magnitude of the peak in ${\chi }^{^{\prime}}$ for different ${H}_{\mathrm{ac}}$ as demonstrated in Fig. 9b for 10 Hz.

**Figure 10:**
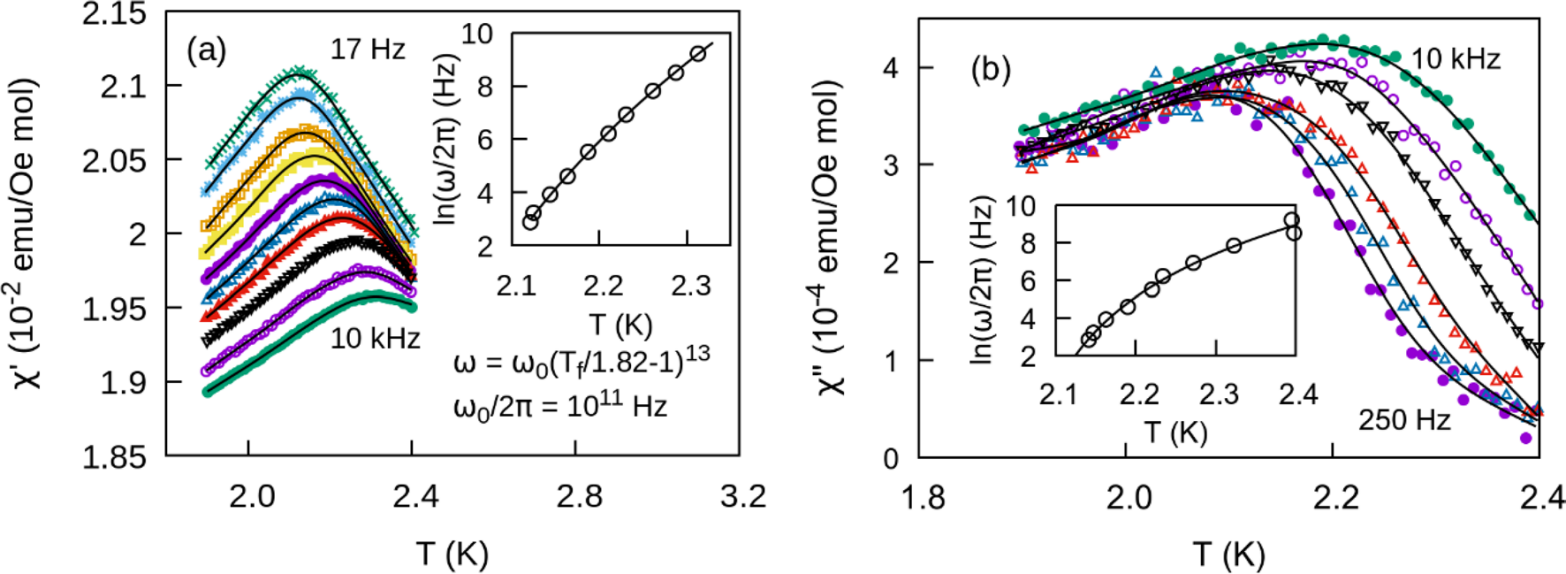
**(a)** ${\chi }^{{\prime}}$ and **(b)** ${\chi }^{{{\prime \prime}}}$ versus temperature. Insets are fits to Eq. (5). ${H}_{ac}$ varies as shown in Fig. 9a.

Susceptibility results for a varying ${\mathrm{H}}_{\mathrm{ac}}$ for the $x=0$ end member are shown in Fig. 10a,b. In this more comprehensive analysis, we find that the dynamical exponents and the spin-flip times fall within the uncertainty of the first set of measurements. These results reinforce our conclusions that the magnetic properties of the solid solution ought to be interpreted as either an insulating spin glass or cluster glass. We also performed a second series of fits using the inflection point of the imaginary part of the susceptibility ${\left(\frac{d{\chi }^{{{\prime \prime}}}}{dT}\right)}_{\mathrm{max}}$ (Table 4). Spin flip times are longer than those derived from ${\chi }^{{\prime}}$ and range from ${10}^{-8}$ to ${10}^{-11}$ s, while $z\upsilon$ values tend to be smaller ranging from 5 to 13. A similar discrepancy between the parameters derived from ${\chi }^{^{\prime}}$ and ${\chi }^{{{\prime \prime}}}$ is observed, for instance, in the insulating spin glass La_0.9_Sr_0.1_CoO_3_^44^. Nevertheless, the dynamic scaling analysis confirms the general observation that the majority of the solid solution most closely resembles that of either a cluster glass or insulating spin glass.

**Table 4:** Same parameters as those in Tables 2 and 3 using the inflection point in ${\upchi }^{\mathrm{^{\prime}}\mathrm{^{\prime}}}$ as the indicator of ${\mathrm{T}}_{\mathrm{f}}$. ${H}_{ac}$ is varied as indicated in Fig. 9a.

x	$K$	${\tau }_{0}$ $(\mathrm{s})$	${T}_{sg} (\mathrm{K})$	$z\upsilon$
0	0.045	${10}^{-8\pm 0.5}$	2.05 $\pm$ 0.03	5 $\pm$ 1
0.4	0.043	${10}^{-9\pm 0.4}$	2.37 $\pm 0.0$ 4	7 $\pm$ 1
0.6	0.037	${10}^{-11\pm 0.5}$	2.4 $\pm$ 0.2	13 $\pm$ 5
0.8	0.033	${10}^{-11\pm 0.6}$	2.53 $\pm$ 0.09	10 $\pm$ 2
1.0	0.033	${10}^{-10\pm 0.8}$	2.6 $\pm$ 0.1	7 $\pm$ 3

**Figure 11:**
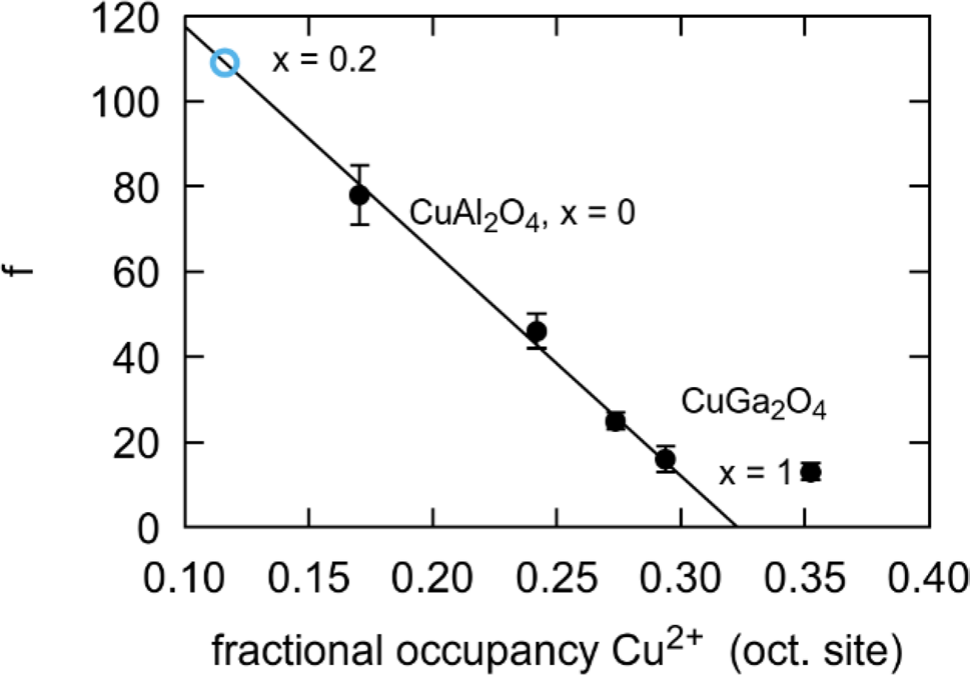
Frustration versus Cu^2+^ occupancy of the octahedral sites. Open blue circle indicates extrapolated value for $x=0.2$.

With an estimate for ${T}_{sg}$ reported in Table 3, we can now estimate how the magnetic frustration evolves with composition in the CuAl_2(1-x)_Ga_2x_O_4_ system. We find a negative linear correlation between the frustration and the occupancy of the octahedral site as shown in Fig. 11. The trend departs from linearity for the $x=1$ member of the solution. Using the occupancy obtained for $x=0.2$, we extrapolate out that $f\sim 110$, and hence ${T}_{sg}\sim 1.5$ K. This temperature is consistent with the fact that we are unable to detect a freezing transition at the low temperature limit of 1.9 K for our apparatus.

## Summary and discussion

We have investigated the magnetic and structural properties of the solid solution CuAl_2(1−x)_Ga_2x_O_4_. Our structural analyses show that the spin 1/2 Cu^2+^ ions favor the tetrahedral sites for CuAl_2_O_4_ ($x=0, \eta =0.38$) and generally redistribute toward the octahedral sites for $x>0.2$. This is similar to the general trend observed for CoAl_2(1−x)_Ga_2x_O_4_^20^ with the larger Ga atom increasing the bond length, leading to a decrease in ${\Theta }_{\mathrm{CW}}$ indicating a decrease in magnetic exchange interactions. However, unlike the Co solid solution, we do not observe a purely paramagnetic phase at $x=0$ due to intersite mixing which results in glassy dynamics spanning the observable portion of the solution.

When detectable via our apparatus, all members of the solution display a bifurcation in the FC-ZFC magnetization data below 3 K with a decrease in transition temperature as a function of applied field. In all cases the FC susceptibility curve continues to increase below the bifurcation temperature suggesting a portion of the spins not participating in the glassy dynamics. This is consistent with the interpretation suggested by Cho et al.^13^ for CuAl_2_O_4_ that a dynamic and glassy phase coexist with the glassy phase corresponding to the degree of site disorder. Further, the applied magnetic field dependent bifurcation temperature deviates from the mean field AT line. We observe similar AT-like behavior in ac susceptibility measurements in the presence of an external dc field. Prior investigation for the $x=1$ member includes modeling that assumes a Heisenberg magnetic system^16^, therefore separating out whether there is an additional GT transition is a topic for further research. Other evidence of spin glass behavior includes slow relaxation dynamics for even members of the solid solution ($x\ge 0.4)$, and memory effects for $x=0.8$.

We have also shown that the ac susceptibility reveals typical glassy behavior, with a peak in ${\chi }^{^{\prime}}(T)$ and an inflection in ${\chi }^{{{\prime \prime}}}(T)$ shifting to lower temperatures with decreasing frequency. The frequency dependent shift fits to a dynamical phase transition typical for spin glasses. Parameters derived from this model fit within ranges for both insulating spin glasses and cluster glasses, although with the higher spin flip times reported in Table 3 we lean towards an insulating spin glass. Therefore, we believe the best interpretation of our solid solution from this analysis is that of a spin glass-like system shifting from a dilute diamond lattice to a dilute pyrochlore as Al is replaced with Ga^13,45^, with weaker frustration as Cu^2+^ shifts to the pyrochlore lattice and bond length increases.

The ac susceptibility results are interesting to compare to a similar A-site spinel FeAl_2_O_4_ ($s=2$) in light of a proposed magnetic phase diagram as a function of $\eta$ ^46^. This diagram is based on CoAl_2_O_4_ and shows an overlapping region separating a spin liquid regime at low values of $\eta$ and spin glass region at higher values. In the case of FeAl_2_O_4_ the ac susceptibility peak does not shift with changing frequency. Hence, assuming a similar phase diagram, the compound is interpreted as residing near the spin liquid/spin glass boundary^47^. By contrast, our results add weight to the idea that the $x=0$ and the $x>2$ portions of the solid solution are either closer to or reside within the spin glass portion of the diagram. $\mu SR$ measurements of the entire solution would reveal the degree to which any additional non-glassy dynamic spin fluctuation state persists and evolves for $x>0$.

Finally, we have obtained frustration values for the even members of the solution. Frustration values greater than 10 are considered large^8^, and are therefore of interest in accessing highly degenerate quantum ground states. A value of $f=67$ and a ${T}_{\mathrm{sg}} \sim 2$ K have been reported by Nirmala et al*.* ^12^ for $x=0$, slightly lower than the values of 69–78 ( ${T}_{\mathrm{sg}}\sim 1.82$ K) we achieved for a sample with the same chemistry. We note a linear trend in magnetic frustration associated with the relocation of the Cu ion as Al is replaced with Ga. Extrapolating from this trend, we predict a frustration value of ~ 110 for $x=0.2$ and a ${T}_{\mathrm{sg}}$~1.5 K. Such a high frustration value makes this instance of the solid solution a candidate for additional investigation as a potential quantum spin glass or spin liquid.

## Experimental details

### Synthesis

The solid solution CuAl_2(1−x)_Ga_2x_O_4_ ($x = 0$ to $1$ in steps of 0*.*1) was created by mixing stoichiometric amounts of CuO, Al_2_O_3_, and Ga_2_O_3_, and allowing them to react at $950^\circ$ C for 100 h in atmosphere in alumina crucibles. The reactants were supplied by Alfa Aesar and possess a purity $\ge 99.99 \%$. These smaller batches (< 1 g) were used for high-resolution structural analysis via synchrotron diffraction and magnetic analysis. A second set of samples (> 4 g per sample), with compositions corresponding to increments of $x=0.2$, were synthesized at $1000^\circ$ C for 20 h. These samples were used for our neutron diffraction studies. In all cases, phase purity was initially assessed via X-ray powder diffraction (Bruker D8 Discover, Cu K_α_ = 1.5408 Å). Trace amounts of CuO and GaO impurities were indexed and included in Rietveld refinement via the Fullprof suite^48^.

### Structural characterization

High-resolution synchrotron X-ray powder diffraction data were collected at room temperature ($295$ K) at the Advanced Photon Source at Argonne National Laboratory using beamline 11-BM ($\lambda =0.412826 \mbox {\normalfont \AA}$). Neutron powder diffraction measurements were performed at Oak Ridge National Laboratory using two complementary techniques: time of flight data were collected on the BL-11A POWGEN instrument at the Spallation Neutron Source and constant-wavelength neutron powder diffraction data were collected at the High-Flux Isotope Reactor facility (HFIR) using HB-2A^49^. Rietveld refinements were performed on all collected data using the Fullprof refinement software suite ^48^.

### Magnetic characterization

Both ac and dc magnetic measurements were performed with an Evercool Physical Property Measurement System (PPMS, Quantum Design) using the vibrating sample magnetometer (VSM) and ac measurement system (ACMS II) options, respectively. Parameters particular to various magnetic measurements performed in this study are discussed in the appropriate Results subsections.
